# Solomonsterol A, a Marine Pregnane-X-Receptor Agonist, Attenuates Inflammation and Immune Dysfunction in a Mouse Model of Arthritis

**DOI:** 10.3390/md12010036

**Published:** 2013-12-24

**Authors:** Andrea Mencarelli, Claudio D’Amore, Barbara Renga, Sabrina Cipriani, Adriana Carino, Valentina Sepe, Elisa Perissutti, Maria Valeria D’Auria, Angela Zampella, Eleonora Distrutti, Stefano Fiorucci

**Affiliations:** 1 Department of Experimental and Clinical Medicine, University of Perugia, Gambuli Street, S. Andrea delle Fratte, Perugia 06132, Italy; E-Mails: andrea.mencarelli@unipg.it (A.M.); barbara.renga@unipg.it (B.R.); sabrina.cipriani@unipg.it (S.C.); adriana.carino@hotmail.it (A.C.); stefano.fiorucci@unipg.it (S.F.); 2 Department of Pharmacy, University of Naples “Federico II”, D. Montesano Street, 49, Naples 80131, Italy; E-Mails: valentina.sepe@unina.it (V.S.); elisa.perissutti@unina.it (E.P.); madauria@unina.it (M.V.D.A.); 3 University and City Hospital of Perugia, Perugia 06100, Italy; E-Mail: eleonoradistrutti@katamail.com

**Keywords:** marine sponge, *Theonella swinhoei*, rheumatoid arthritis, inflammation, pregnane X receptor (PXR), solomonsterol A

## Abstract

In the present study we provide evidence that solomonsterol A, a selective pregnane X receptor (PXR) agonist isolated from the marine sponge *Theonella swinhoei*, exerts anti-inflammatory activity and attenuates systemic inflammation and immune dysfunction in a mouse model of rheumatoid arthritis. Solomonsterol A was effective in protecting against the development of arthritis induced by injecting transgenic mice harboring a humanized PXR, with anti-collagen antibodies (CAIA) with beneficial effects on joint histopathology and local inflammatory response reducing the expression of inflammatory markers (TNFα, IFNγ and IL-17 and chemokines MIP1α and RANTES) in draining lymph nodes. Solomonsterol A rescued mice from systemic inflammation were assessed by measuring arthritis score, CRP and cytokines in the blood. In summary, the present study provides a molecular basis for the regulation of systemic local and systemic immunity by PXR agonists.

## 1. Introduction

A class of ligand activated regulatory factors termed nuclear receptors (NRs) has been identified as critical biological guidelines for many biological processes including fine-tuning regulation of innate and adaptive immunity and offers the prospect of multiple targeting. Pregnane X Receptor (PXR) is a member of the NR superfamily acting as a major endo- and xeno-biotics sensor. PXR is mainly expressed in the gastrointestinal tract and liver [1,2,3,4], and to a lesser extent in the kidney and leukocytes [5,6,7,8]. PXR regulates gene expression by forming heterodimers with RXR and subsequently binding to xenobiotic responsive elements present in the promoter regions of genes encoding drug-metabolizing enzymes and transporters (*i.e.*, CYP3A4, SULT2A1, ABCC2). In addition to its role in regulating xenobiotic metabolism, PXR exerts immunomodulatory and anti-inflammatory activities by inhibiting the function of NF-κB [5,9,10], a transcription factor which regulates the production of many genes associated with both innate and adaptive immunity such as cytokines, chemokines, adhesion proteins and stress response genes. The interaction of NF-κB with PXR leads to a reciprocal regulation of these two factors with PXR acting as a negative regulator of NF-κB activity [11,12]. Moreover, PXR activation in T cells inhibits proliferation, reduces Interferon (IFN)-γ expression and thwarts MEK1/2 and NF-κB signaling [5].

**Figure 1:**
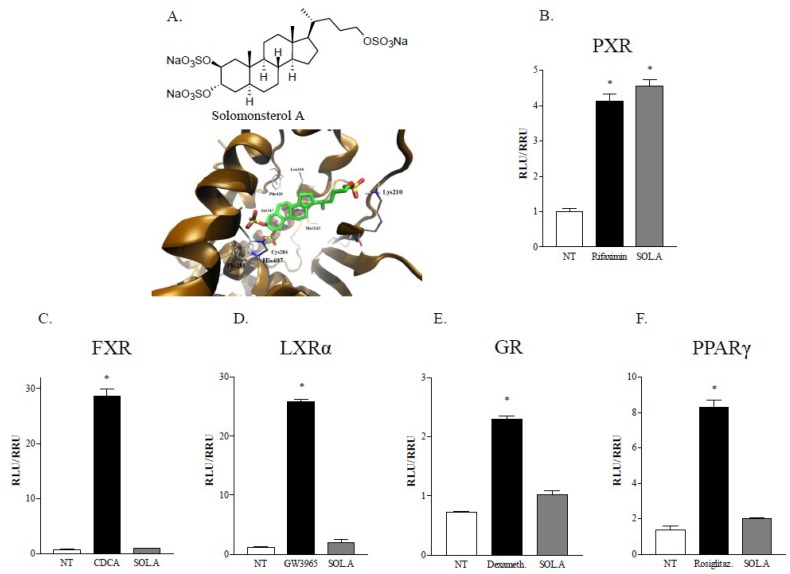
Sulfated sterol solomonsterol A is a selective ligand for the nuclear receptor pregnane X receptor (PXR). (**A**) Solomonsterol A chemical structure and PXR-LBD-solomonsterol A docking picture. (**B**–**F**) Transactivation assay performed in HepG2 cells to evaluate solomonsterol A capability to induce several nuclear receptors activity. Cells were transfected, as reported in the experimental section, for (**B**) PXR, (**C**) FXR, (**D**) LXRα, (**E**) GR and (**F**) PPARγ mediated transactivation and primed with solomonsterol A 10 μM for 18 h. Rifaximin (10 μM), CDCA (10 μM), GW3965 (10 μM), Dexamethasone (1 μM) and Rosiglitazone (200 nM) were used as positive control. The values are expressed as mean ± SD. (* *p* < 0.05, compared to *not treated* cells; *N* = 4).

Recently, we have reported the isolation of several PXR ligands from marine sources [13,14,15,16,17,18]. Among these, solomonsterol A (Figure 1), a sulfated sterol extracted from the marine sponge *Theonella swinhoei*, was found to be the first example of a marine potent human PXR agonist boosting the receptor activity by 4−5 fold in transactivation assays [19] and stimulating the expression of PXR target genes CYP3A4 and MDR1 in a human hepatocyte cell line.

From a structural point of view, key features of solomonsterol A are the presence of a truncated C24 side chain, and three sulfate groups at C2, C3 and C24 (Figure 1A). Through docking and medicinal chemistry approaches [19,20], we demonstrated that the three sulfate groups in the molecules contribute to accommodate the steroid nucleus in PXR-LBD (Figure 1A) acting as key points of interaction with three polar aminoacids, Lys210 (electrostatic interactions with sulfate group on the side chain), Ser247 and His407 (electrostatic interactions with the two sulfate group on ring A).

Moreover, we have shown that solomonsterol A modulates LPS induced NF-κB DNA binding activity in THP-1 cells and that PXR agonism exerted by administering solomonsterol A in transgenic mice expressing human PXR results in IL-10 stimulation [21].

Rheumatoid arthritis (RA) is a chronic autoimmune disease involving synovial inflammation and adjacent cartilage and bone destruction. RA causes progressive disability associated with early mortality primarily reflecting vascular co-morbidity. RA is driven by dysregulated adaptive and innate immune pathways [22,23,24,25]. Cytokine inhibitors (e.g., anti-TNFα), B-cell depletion and T-cell blockade are components of current standard of disease. Partial, transient, or non-response is common, however, and clinical or radiographic remission is rarely sustained; significant unmet clinical need remains. The prospect of targeting multiple pathways simultaneously is attractive to optimize the neutralization of complex effector immune pathways.

The observation that patients affected by osteoarthritis show lower expression of several NRs, including the PXR [26], suggests that re-induction of PXR expression could exert beneficial effects in RA treatment. In this study we have investigated the anti-inflammatory activity of solomonsterol A in an experimental model of systemic inflammation, using the CAIA (Anti-type II collagen antibody-induced arthritis) model, a widely used model to study human RA.

Results of present study suggest that targeting PXR might be of relevance in treating systemic inflammation.

## 2. Results

### 2.1. Sulfated Sterol Solomonsterol A Is a Selective Human PXR Agonist

We examined whether solomonsterol A is a selective ligand for PXR. To ascertain this, we investigated whether solomonsterol A interacts with a panel of nuclear receptors including PXR, FXR, LXRα, GR and PPARγ in a transactivaton assay in HepG2 cells. As illustrated in Figure 1, panels B–F, solomonsterol A at the concentration of 10 μM effectively transactivates PXR but failed to transactivate the other nuclear receptors, thus indicating that this agent is a selective PXR agonist.

**Figure 2:**
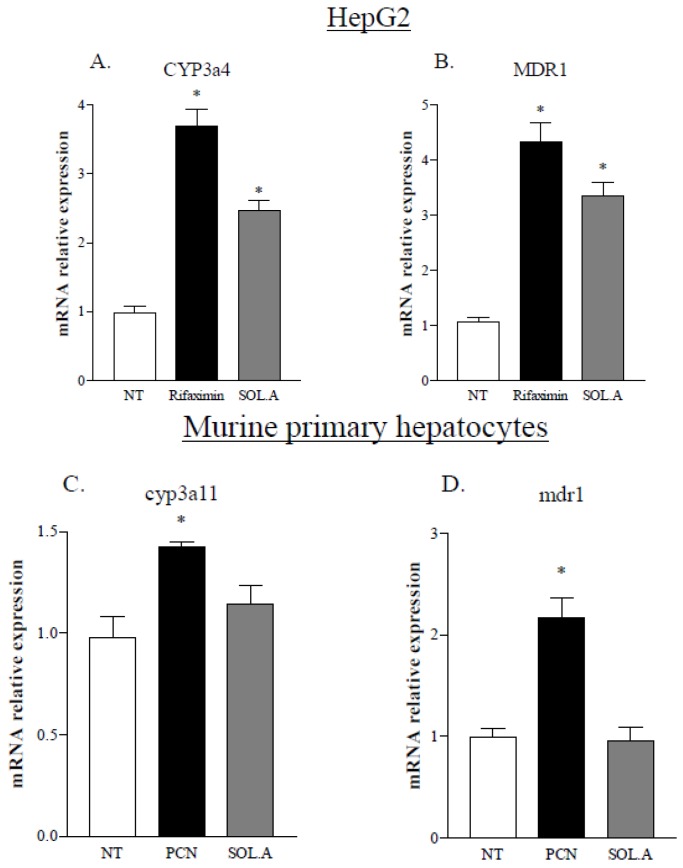
Solomonsterol A is a selective human PXR agonist. (**A**,**B**) Real-time PCR analysis of the human PXR target genes CYP3A4 and MDR1 in HepG2 cells primed with rifaximin 10 μM and solomonsterol A 10 μM for 18 h. (**C**,**D**) Real-time PCR analysis of the murine PXR target genes *cyp3a11* and *mdr1* in primary hepatocytes stimulated *ex vivo* with 10 μM pregnenolone 16α-carbonitrile (PCN), a murine PXR agonist, and solomonsterol A 10 μM for 18 h. The values are expressed as mean ± SD. (* *p* < 0.05, compared to *not treated* cells; *N* = 3).

To further investigate on the specificity of solomonsterol A as a PXR agonist across species, we have tested whether solomonsterol A activates the murine PXR. To this end we assessed the expression of several PXR target genes in a human hepatocarcinoma cell line, HepG2 cells, and in murine primary hepatocytes, isolated from the liver of wild type C57BL/6 mice, primed with solomonsterol A, 10 μM. Rifaximin and pregnenolone 16α-carbonitrile (PCN) were used as positive controls for human and murine PXR. As shown in Figure 2, while solomonsterol A increased the expression of CYP3A4 and MDR1 in HepG2 cells (human PXR), this treatment failed to modulate the expression of the PXR target genes *cyp3a11* and *mdr1* in murine primary hepatocytes (murine PXR). All together, these results suggest that solomonsterol A is a selective human PXR agonist.

### 2.2. Solomonterol A Administration Reduces Clinical and Local Signs of Arthritis

**Figure 3:**
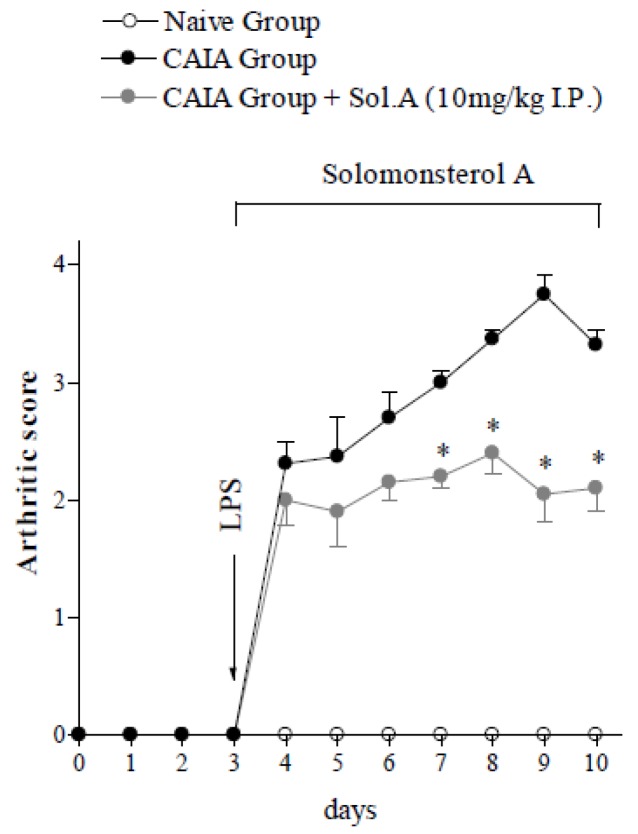
The PXR agonist solomonsterol A protects against rheumatoid arthritis development in PXR transgenic mice expressing the human PXR gene. Treatment with solomonsterol A significantly inhibited development and progression of CAIA. Arthritic score values as reported as mean ± SD (* *p* < 0.05 compared to *CAIA mice group*; *N* = 5).

We then examined whether solomonsterol A was effective in reducing systemic inflammation in a mouse model of rheumatoid arthritis. To this end, we used the CAIA model, a variant of CIA (collagen II-induced arthritis) model. In this model, joint inflammation is induced by administering mice with a cocktail of five monoclonal antibodies to type II collagen. Antibodies were administered intravenously on day 0, followed by an intraperitoneal injection of LPS (50 μg/mouse) on day 3. LPS acts in a synergistic fashion with auto-antibodies to boost the onset of arthritis in mice. The CAIA arthritis model is characterized by a rapid induction of joint inflammation (arthritis develops on day 4 and reaches its peak on days 7–8), is strain independent and highly reproducible. To investigate the function of PXR agonism in this model, CAIA was induced in C57BL/6 transgenic mice expressing the human PXR gene. Animals were then treated with vehicle or solomonsterol A as indicated in Figure 3 [19,21].

Clinical arthritis scores were recorded from day 0 and graded on a scale of 0–4 for the extent of paws edema, erythema, swelling and deformation, as described in the experimental section. First clinical signs of arthritis were observed on day 4 (one day after LPS administration) and the disease peak was achieved on day 9 when 100% of mice developed the clinical signs of the disease. As reported in Figure 3, mice treated with solomonsterol A developed an attenuated form of inflammation as measured by assessing the arthritic index with a 30% reduction of the arthritic score in solomonsterol A-treated group on day 7 compared to CAIA-group (Figure 3, *p* < 0.05; *N* = 5).

**Figure 4:**
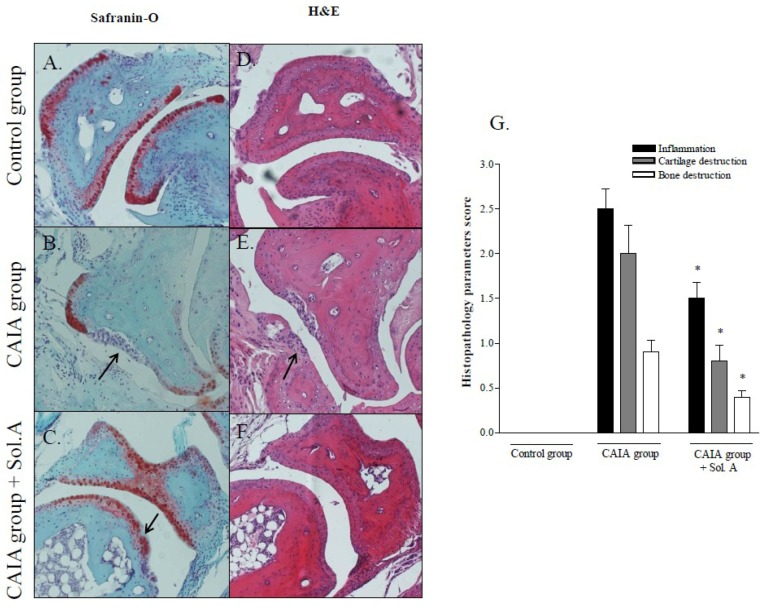
Solomonsterol A administration prevents cartilage joints erosion in arthritic mice. Safranin-O (**A**–**C**) and Hematoxylin and Eosin (**D**–**F**) histological staining of joints of control, CAIA and CAIA + solomonsterol A (Sol.A) mice. The microphotographs represent an individual animal and are representative of five animals per group. The arrows indicate areas of inflammatory cells infiltration, cartilage and bone disruption. (**G**) Histopathological scores for joint inflammation, cartilage and bone destruction. The values are expressed as mean ± SD (* *p* < 0.05 compared to *CAIA group*; *N* = 5).

The histopathology analysis demonstrates that joints from arthritic mice had mild to moderate infiltration by inflammatory cells and cartilage disruption with minimal bone erosion, as indicated by Safranin-O and H&E staining (Figure 4, panels B, E and G); administering CAIA mice with solomonsterol A resulted in a robust attenuation of the prototypical changes in the CAIA model (Figure 4, panels C, F and G).

### 2.3. Solomonsterol A Administration Reduces Systemic Signs of Arthritis

**Figure 5:**
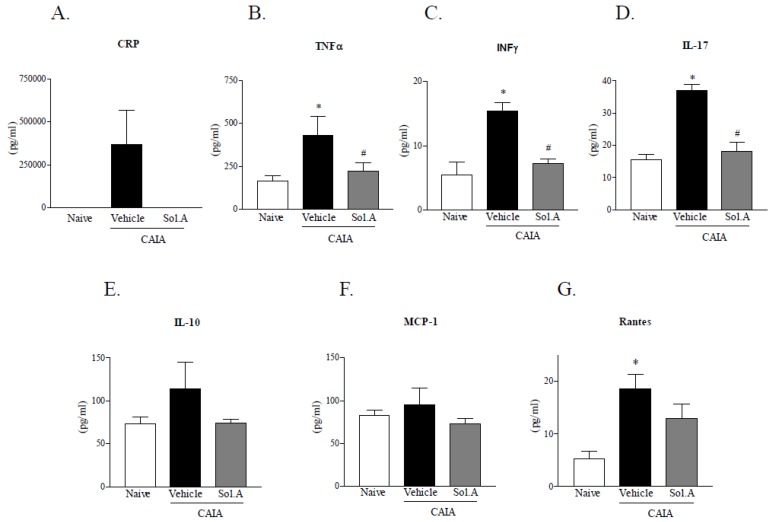
Solomonsterol A ameliorates systemic signs of arthritis. Plasma levels of (**A**) C-Reactive Protein (CRP); (**B**) Tumor Necrosis Factor α (TNFα); (**C**) Interferon γ (INFγ); (**D**) Interleukin 17 (IL-17); (**E**) Interleukin 10 (IL-10); (**F**) Monocyte Chemoattractant Protein 1 (MCP-1); (**G**) Rantes. The values are expressed as mean ± SD. (* *p* < 0.05, compared to *untreated group*; # *p* < 0.05, compared to *CAIA mice group*; *N* = 5).

Plasma samples collected from control, CAIA and CAIA*-*solomonsterol A groups were used to measure the levels of C-Reactive Protein (CRP), Tumor Necrosis Factor α (TNF-α), Interferon γ (INF-γ), Interleukin 17 (IL-17), Interleukin 10 (IL-10), Monocyte Chemoattractant Protein 1 (MCP-1) and Rantes. As shown in Figure 5, arthritis induced by antibodies to type II collagen resulted in increased levels of TNF-α, INF-γ, IL-17 and Rantes (* *p* < 0.05; *N* = 5). CRP levels were increased in three of the five animals of CAIA group. Administration of solomonsterol A to arthritic mice reduced TNF-α, INF-γ and IL-17 and abrogated completely changes in CRP plasma levels induced by CAIA (Figure 5, panels A–D, # *p* < 0.05; *N* = 5). No significant changes were detected in plasma levels of IL-10 and MCP-1 between three groups (Figure 5, panels E and F).

### 2.4. PXR Agonism Abrogates Arthritic Profile of DLN Cells Induced by CAIA Treatment

In RA patients, the delivery of cytokines to lymph nodes increases due to joint inflammation [27]. We have therefore decided to investigate draining lymph nodes (DLN) cells to characterize their proliferation activity, Th17, Th1 and Treg cytokines production and chemokine release in basal condition and after polyclonal stimulation of mouse T cells using concanavalin A (ConA) *in vitro*.

**Figure 6:**
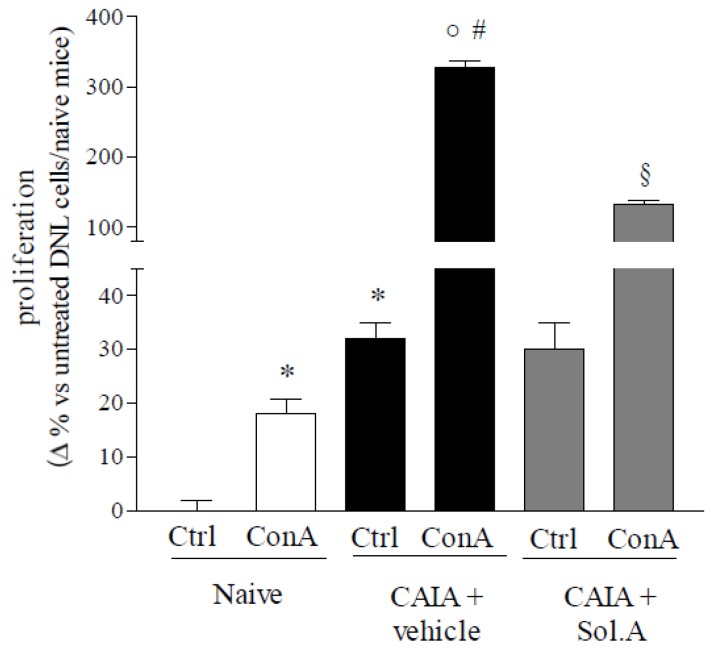
*In vivo* PXR agonism results in a reduced proliferation rate of DLN cells after ConA stimulation *ex vivo*. Proliferation measured in DLN cells after 36 h of culture in basal and activated condition by concanavalin A (ConA) stimulation (2 µg/mL); proliferation rate are expressed as delta of absorbance compared to untreated cells obtained from control group. The values are expressed as mean ± SD. (* *p* < 0.05 compared to *Naive control group*; o *p* < 0.05 compared to *Naive + ConA group*; # *p* < 0.05 compared to *CAIA control group*; § *p* < 0.05 compared to *CAIA + ConA group*; *N* = 3).

As reported in Figure 6, DLN cells obtained from arthritic mice, in basal condition, showed an increased proliferation rate (~30%) compared to control DLN cells (* *p* < 0.05; *N* = 5); moreover, DLN cells from CAIA group was more sensitive to ConA stimulation compared to DLN cells obtained from intact animals. Priming with ConA boosted the proliferation rate of DLN cells obtained from CAIA mice by 18-fold (Figure 6; “ο” and “#” *p* < 0.05; *N* = 5). In contrast, *in vivo* PXR agonism resulted in a significant reduction of the proliferation rate of DLN cells exposed to ConA (Figure 6; “§” *p* < 0.05; *N* = 5).

**Figure 7:**
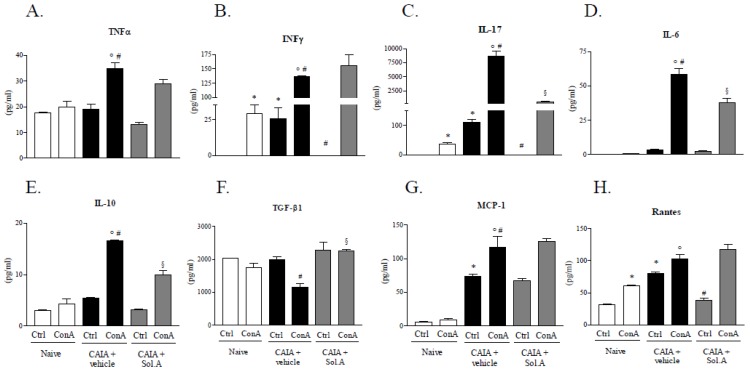
*In vivo* solomonsterol A administration partially abrogates the arthritic profile of DLN cells in CAIA mice. Cytokines and chemokines levels in DLN cells after 36 h of culture in basal and activated condition by concanavalin A (ConA) stimulation (2 µg/mL). (**A**) TNFα; (**B**) INFγ; (**C**) IL-17; (**D**) IL-6; (**E**) IL-10; (**F**) TGF-β1; (**G**) MCP-1; (**H**) Rantes. The values are expressed as mean ± SD. (* *p* < 0.05 compared to *Naive control group*; o *p* < 0.05 compared to *Naive + ConA group*; # *p* < 0.05 compared to *CAIA control group*; § *p* < 0.05 compared to *CAIA + ConA group*; *N* = 3).

To investigate local anti-inflammatory effects of solomonsterol A, we investigated the cytokines and chemokines pattern of T cells obtained from DLNs of the three experimental groups and TNF-α, INF-γ, IL-17, IL-6, IL-10, Transforming Growth Factor-β1 (TGF-β1), MCP-1 and Rantes levels were evaluated. In basal condition, DLN cells obtained from arthritic mice released more INF-γ, IL-17, MCP-1 and Rantes in comparison to the same cell population of healthy animals (Figure 7, panels B, C, G and H; “*” *p* < 0.05; *N* = 5). Polyclonal stimulation of T cells from Control group mice resulted in significant changes only in INF-γ, IL-17 and Rantes release (Figure 7, panels B, C and H; “*” *p* < 0.05; *N* = 5) whereas, after ConA stimulation, DLN cells from CAIA group mice showed an increased release of all inflammatory mediators analyzed (Figure 7, panels A–E and G; “ο” and “#” *p* < 0.05; *N* = 5), with the exception of Rantes (no significant change) and TGF-β1, that were significantly reduced (Figure 7, panel F; “#” *p* < 0.05; *N* = 5).

*In vivo* solomonsterol A administration, *per se*, was able to reduce the Rantes release and completely abrogated INF-γ and IL-17 release in DLN cells isolated from CAIA+solomonsterol A group, compared to T cells obtained from arthritic mice (Figure 7, panels C and D; “#” *p* < 0.05; *N* = 5). Finally, draining lymph node cells from arthritic mice administered with solomonsterol A and triggered with ConA, showed a diminished release of IL-17, IL-6 and IL-10 when compared with T cells from arthritic mice stimulated with ConA (Figure 7, panels C–E; “§” *p* < 0.05; *N* = 5) and, simultaneously, *in vivo* solomonsterol A treatment abrogated *in vitro*, lowering the release of TGF-β1, which was reverted to basal levels (Figure 7, panel F; “§” *p* < 0.05; *N* = 5).

These data demonstrate that *in vivo* PXR agonism was able to partially abrogate the arthritic profile of DNL cells evoked by administration of antibodies to type II collagen, observed *in vitro* after polyclonal stimulation.

### 2.5. Solomonsterol A Modulates PXR Target Genes in the Liver

**Figure 8:**
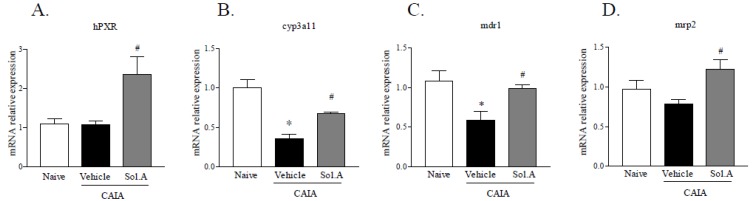
Effects of solomonsterol A (Sol.A) on regulation of liver expression of PXR and PXR-target genes in response to CAIA. Real-time PCR analysis of (**A**) hPXR, (**B**) *cyp3a11*, (**C**) *mdr1* and (**D**) *mrp2*. The values are expressed as mean ± SD. (* *p* < 0.05 compared to Naive control group; # *p* < 0.05 compared to CAIA mice group; *N* = 5).

Since PXR is highly expressed in the liver, where it plays a canonical role as master gene regulating the activity of a variety of genes involved in xeno- and endo-biotic metabolism, we decided to evaluate hepatic expression of PXR, *cyp3a11*, *mdr1* and *mrp2*, three well-known pregnane X receptor target genes, after the induction of the arthritic disease by CAIA treatment and the administration of solomonsterol A. As reported in Figure 8A, liver PXR gene expression was unchanged in hPXR mice exposed to CAIA, and treatment with solomonsterol A caused a two-fold induction of PXR expression. Analysis of PXR target genes showed a significant reduction of *cyp3a11* and *mdr1* expression in CAIA group mice (Figure 8, panels B and C; “*” *p* < 0.05; *N* = 5), while no change in *mrp2* expression was observed between Control group and CAIA group mice. Noteworthy, solomonsterol A administration resulted in a strong induction of all three PXR target genes in comparison with mice of CAIA group (Figure 8, panels B, C and D; “#” *p* < 0.05; *N* = 5), as a consequence of the robust induction of PXR gene observed in mice administered with PXR agonist.

## 3. Experimental Section

### 3.1. Animals

Humanized (h)PXR mice, 8–10 weeks of age, were provided by Dr. Frank J. Gonzalez (Laboratory of Metabolism, Centre for Cancer Research, National Cancer Institute, National Institutes of Health, Bethesda, MD, USA) [28]. The hPXR mice express the human PXR in the mice PXR-null background. All mice were housed in a temperature controlled room (22 °C) and photoperiods (12 h light–12 h dark cycle), and had free access to food and water. Protocols were approved by the University of Perugia Animal Care Committee according to the Italian guideline for care and use of laboratory animals. The ID for this project is #98/2010-B. The authorization was released to Prof. Stefano Fiorucci, as a principal investigator, on May 19, 2010.

### 3.2. Induction of Arthritis: CAIA Mouse Model

Arthritis was induced in C57BL/6 male mice using “Arthrogen-CIA” kit (Chondrex Inc, Redmond, WA, USA) according to the manufacturer’s instructions. Under standardized conditions, mice were divided into three groups (*N* = 5 for group): an untreated group (normal control), a collagen antibody induced-arthritis group (CAIA) and a CAIA *plus* solomonsterol A treated group. Exactly on day 0 we injected 2.5 mg of 5-clone cocktail/mouse by intravenous injection and on day 3 we injected 50 μg/mouse of LPS, by intraperitoneal injection (I.P.). From day 3, one group received systemic administration of solomonsterol A (dissolved in methylcellulose 1%) 10 mg/kg, daily, by intraperitoneal injection. Animals of control and CAIA groups were administered daily with methylcellulose 1% (vehicle) from day 3 until the end of the experiment.

Mice were monitored daily for clinical arthritis scores; each paw received a score as follow: 0 = no visible effects of arthritis; 1 = edema and/or erythema of one digit; 2 = edema and/or erythema of two joints; 3 = edema and/or erythema of more than two joints; 4 = severe arthritis of the entire paw and digits. Arthritic index was calculated by adding all individual paw scores (maximum arthritic index = 16), and then recorded. The group means for the summed scores of all animals were plotted over time [29,30,31].

### 3.3. Biochemical Analyses and Histopathologic Assessment

At the end of the experiment, plasma was collected for C-reactive protein, TNFα, INFγ, IL-17, IL-10, MCP-1 and Rantes determinations by “Bioplex ELISA” kit (Bio-Rad Laboratories s.r.l., Hercules, CA, USA).

For histological analysis, paws were excised, skinned and fixed in 4% buffered formaldehyde and then decalcified in 12% disodium EDTA for 15 days. Tissues were then paraffin embedded, sectioned and Safranin-O and Hematoxylin and Eosin (H&E) stained. The severity of the arthritis in the joint was scored in a blind manner by two independent observers; the three parameters analyzed (joint inflammation, cartilage and bone destruction) were scored on a scale of 0–5 (0 = Normal; 1 = Minimal; 2 = Mild; 3 = Moderate; 4 = Marked; 5 = Severe).

### 3.4. Cell Preparation and Activation

Inguinal and popliteal Draining Lymph Nodes (DLN) cells were extracted from all experimental groups and cultured in 96 plate wells (2 × 10^6^/mL, 250 µL/well; *N* = 6 for group) alone or in combination with concanavalin A (2 µg/mL), for 36 h. Cell proliferation was evaluated by “Cell Counting Kit-8” (Alexis Biochemicals, San Diego, CA, USA) after 3 h of incubation. In another experimental settings, DLN cells were cultured and supernatants collected for cytokines and chemokines production by “Bio-plex ELISA” kit (Bio-Rad Laboratories s.r.l., Hercules, CA, USA).

### 3.5. Isolation and Culture of Murine Primary Hepatocytes

Primary hepatocytes were isolated from C57BL/6 mice anesthetized with pentobarbital sodium solution (50 mg/kg I.P.). In brief, the inferior vena cava was canulated and the liver was first perfused *in situ* with an oxygenated Hank’s Balanced Salt Solution (HBSS) containing EDTA 0.5 mM, pH 8 (5 mL/min, 37 °C for 10 min), followed by perfusion with Dulbecco’s Modified Eagle’s Medium (DMEM) containing collagenase type I 0.8 mg/mL (5 mL/min, 37 °C for 12 min). The liver was removed and gently minced in HBSS; liver cell suspension was filtered with Falcon cell strainers (100 μM) and centrifuged at 50 g for 1 min, then pellet was washed twice with DMEM. Cell viability, determined by trypan blue staining, was generally >85%. Cells were re-suspended in DMEM containing BSA 2% at 1 × 10^6^ cells/mL, spinned 50 g for 1 min, and finally resuspended in DMEM:F12 supplemented with 5% fetal bovine serum, 100 U/mL penicillin/streptomycin, 2 mM l-glutamine, 100 nM insulin and 100 nM Dexamethasone. Primary hepatocytes were plated in 6-wells plates at 3 × 10^5^ cells/plate and cultured at 37 °C with 5% CO_2_. After an initial 4 h attachment period, cultures were washed with phosphate-buffered saline (PBS) and then primed with solomonsterol A 10 μM and pregnenolone 16α-carbonitrile (PCN) 10 μM for 18 h.

### 3.6. HepG2 Cell Culture

HepG2 cells were maintained at 37 °C in E-MEM containing 10% FBS, 1% l-glutamine and 1% penicillin/streptomycin. To value whether solomonsterol A administration regulates PXR target genes expression, serum starved HepG2 cells were stimulated for 18 h with 10 μM rifaximin and 10 μM solomonsterol A.

### 3.7. RNA Extraction and Real-Time PCR

Total RNA was isolated from liver samples of hPXR transgenic mice, from murine primary hepatocytes and from HepG2 cells using TRIzol reagent (Invitrogen, Carlsbad, CA, USA) and reverse-transcribed using random hexamer primers and Super Script-II reverse transcriptase (Invitrogen, Carlsbad, CA, USA). mRNA was quantified by Real-Time quantitative PCR on iCycler apparatus (Bio-Rad Laboratories s.r.l., Hercules, CA, USA) using specific primers: m*Gapdh*: ctgagtatgtcgtggagtctac and gttggtggtgcaggatgcattg; m*Cyp3a11*: tgaaaccaccagtagcacac and ccatatccaggtattccatctcc; m*Mdr1*: caggagcccattctctttga and cgatgaactggtggatgttg; m*Mrp2*: aattccttaaccggggacac and gcgcctcattatccacattt; hGAPDH: gaaggtgaaggtcggagt and catgggtggaatcatattggaa; hPXR: agctggaaccatgctgactt and cacatacacggcagatttgg; hCYP3a4: caagacccctttgtggaaaa and cgaggcgactttctttcatc; hMDR1: gtggggcaagtcagttcatt and tcttcacctccaggctcagt. For quantitative RT-PCR, 10 ng of template was dissolved in a 20 μL solution containing 200 nM of each primer and 10 μL of KAPA SYBR FAST Universal qPCR Kit (KAPA BIOSYSTEMS, Wilmington, MA, USA). All reactions were performed in triplicate, and the thermal cycling conditions were as follows: 2 min at 50 °C, 10 min at 95 °C, followed by 50 cycles of 95 °C for 15 s, 58 °C for 30 s and 72 °C for 30 s. The relative mRNA expression was calculated accordingly with the C*t* method. All PCR primers were designed using the software PRIMER3 [32] using published sequence data obtained from the NCBI database.

### 3.8. Transactivation Assay

For PXR mediated transactivation, 5 × 10^4^ HepG2 cells were plated in a 24-wells plate and transfected, using Fugene HD transfection reagent (Roche Holding AG, Basel, Switzerland), with 75 ng of pSG5-PXR, 75 ng of pSG5-RXR, 100 ng of pGL4.70-Renilla and with 250 ng of the reporter vector containing the PXR target gene promoter (pCYP3A4promoter-TKLuc). To examine solomonsterol A specificity, HepG2 cells were transiently transfected with 250 ng of the reporter vector p(UAS)_5×_-TKLuc, 100 ng of pGL4.70-Renilla and with different vectors (150 ng each) containing the ligand binding domain of various nuclear receptors (LXRα, GR and PPARγ) cloned upstream of the GAL4-DNA binding domain. To investigate FXR mediated transactivation, HepG2 cells were transfected with 75 ng of pSG5-FXR, 75 ng of pSG5-RXR, 100 ng of pGL4.70-Renilla and with 250 ng of the reporter vector containing the FXR response element IR1 (pHSP27-TKLuc).

At 24 h post-transfection, cells were stimulated with 10 μM solomonsterol A and, as positive control for each NR mediated transactivation assay, rifaximin 10 μM, CDCA 10 μM, GW3965 10 μM, dexamethasone 1 μM and rosiglitazone 200 nM (respectively for PXR, FXR, LXRα, GR and PPARγ mediated transactivation). 20 μL of cellular lysates were read using Dual-Luciferase Reporter Assay System (Promega Italia s.r.l., Milan, Italy) according manufacturer specifications using the Glomax20/20 luminometer (Promega Italia s.r.l., Milan, Italy). Luciferase activities were normalized for transfection efficiencies by dividing the Luciferase relative light units (RLU) by Renilla relative lights units (RRU) expressed from cells co-transfected with pGL4.70-Renilla.

### 3.9. Synthesis of Solomonsterol A

Solomonsterol A was prepared in a ten steps protocol starting from commercially available hyodeoxycholic acid (33% overall yield) as previously described [21]. The purity of tested solomonsterol A was determined to be always greater than 95% by analytical HPLC analysis on a Macherey-Nagel Nucleodur 100-5 C18 (10 μm; 10 mm i.d. × 250 mm) column using 32% MeOH/H_2_O (isocratic mode) at a flow rate of 3 mL/min.

Solomonsterol A: white solid; [α]_24_^D^= +4.6 (*c* 0.8, CH_3_OH); HRMS-ESI *m/z* 661.1388 [M − Na]^−^, C_24_H_39_Na_2_O_12_S_3_requires 661.1399. The complete match of optical rotation, NMR and HRMS data of solomonsterol A with that of the natural product [19] secured the identity of the synthetic derivative.

### 3.10. Statistical Analysis

All values are expressed as mean ± S.D. of n values per group. Comparisons of more than two groups were made with a one-way ANOVA with post-hoc Tukey’s test. Comparison of two groups was made using Student’s *t*-test for unpaired data when appropriate. Differences were considered statistically significant if *p* was <0.05.

## 4. Discussion and Conclusions

In the present study we have provided compelling evidence that solomonsterol A, a natural compound obtained from the marine sponge *Theonella swinhoei*, exerts anti-inflammatory activity and attenuates systemic inflammation and immune dysfunction in a mouse model of RA. PXR is a xenosensor and regulates the expression of genes involved in xenobiotics oxidation (e.g., CYPs), conjugation (e.g., UGTs and GSTs) and transport (e.g., MDR1, MRP2 and OATP2) and is involved in the metabolism and elimination of potentially harmful chemicals from the body [33]. PXR and its target genes are critical components of the intestinal barrier function against xenobiotics and bacteria and single-nucleotide polymorphisms in PXR gene associate with a decrease in PXR activity and an increase in susceptibility to Crohn’s disease, a chronic self-destructive disorder of the intestine [34]. PXR is a major regulator of CYP3A4, a human homolog of rodent *cyp3a11*, and mainly expressed in the adult entero-hepatic system [11]. It is estimated that CYP3A4 is responsible for the metabolism of almost 50% of drugs used today.

We and others have previously shown that activation of intestinal PXR by rifaximin, a poorly absorbable rifampicin derivative, activates the human PXR and effectively rescues from intestinal inflammation in transgenic mice harboring the human PXR gene [9,10]. Since PXR is predominantly expressed in the entero-hepatic system, the relevance of targeting PXR in the treatment of systemic inflammation, however, remain unclear.

Solomonsterol A is a potent and highly selective agonist to human PXR [19,21]. Thus not only exposure of human hepatocytes to this agent results in a robust transactivation of PXR in the luciferase-gene reporter assay, but when administered orally to PXR transgenic mice, solomonsterol A was as effective as rifaximin in protecting against development of colon inflammation and immune dysfunction in chemical models of colitis. Similarly to rifaximin, solomonsterol A inhibited NF-κB activity *in vitro* and reduced the production of inflammatory cytokines including signature cytokines such as TNF-α, IFN γ and IL-6 and chemokines such as MIP1α. The potential of solomonsterol A for a wide anti-inflammatory activity is also indicated by the reduction of activity of inflammatory mediators such as MPO along with a robust attenuation of inflammatory response at histopathology level [19,21].

PXR gene expression is highly compartmentalized in the entero-hepatic tissue, thus, to date it is still unclear whether PXR agonists might have a pharmacological relevance in modulating systemic inflammation [2]. In the current study we have provided evidence that administration of solomonsterol A to transgenic mice harboring a humanized PXR activates systemic PXR and attenuates systemic inflammation. Thus, solomonsterol A increased the liver expression of *cyp3a11*, *mdr1* and *mrp2*, three PXR regulated target genes [19,21]. Notably, our data demonstrate that expression of these genes in the liver is robustly down-regulated in the CAIA model [26]. The liver expression of *cyp3a11* and *mdr1* was reduced by approximately 50% in the CAIA model, highlighting that RA impairs liver metabolism of xeno- and endo-biotic and might result in widespread dysregulation of PXR regulated activities. Interestingly, these effects were reversed by solomonsterol A. Further on, solomonsterol A increased the liver expression of PXR. Taken together, these data illustrate that solomonsterol A is absorbed from the intestine and reaches the systemic circulation modulating the expression of PXR and its target genes in the liver.

An important observation we have made is that administering PXR transgenic arthritic mice with solomonsterol A protects against development of immune dysfunction and attenuates joint histopathology changes that are prototypical of the CAIA model. CAIA mice have proved to be a useful RA model possessing many cellular and humoral immunity features found in human RA [30]. Treatment with solomonsterol A reduced the degree of joint damage by decreasing the expression of inflammatory mediators. The most interesting finding was that solomonsterol A significantly suppresses the development of arthritis in CAIA mice, which indicates a prophylactic effect of PXR agonist against arthritis onset.

Analysis of inflammatory mediators in the systemic circulation demonstrates that solomonsterol A effectively counteracts the generation of signature markers of RA including TNFα, IFNγ, IL-17 and Rantes and that these changes correlate with a robust reduction of CRP, a clinically validated marker of RA activity [29,30]. The potential for immune regulation of solomonsterol A was further validated by investigating the effect of this PXR ligand on proliferation and activation of immune cells obtained from the draining lymph nodes. Of relevance, not only solomonsterol A attenuates cell proliferation caused by concanavalin A, but it also counter-regulates a number of effector functions as demonstrated by attenuation of generation of inflammatory mediators including TNFα, IFNγ, IL-17, IL-6. In addition, exposure to solomonsterol A caused a slight but significant increase in TGFβ. Because TGFβ is a counter-regulatory molecule in inflammation, the effect that solomonsterol A exerts on this mediator might have relevance in explaining the immune-regulatory activity of this marine PXR ligand [35].

These immune regulatory effects translated in a robust attenuation of histopatological changes caused by CAIA. Indeed, as shown by histopathology analysis and scoring, solomonsterol A effectively reduces immune cells recruitment and cartilage destruction usually occurring in the model, further highlighting the potential of solomonsterol A in the treatment of systemic inflammation.

The present study has some potential limitations. PXR is highly expressed in the liver and regulates a number of genes involved in xenobiotic metabolism. Thus, the activation of PXR holds the risk for development of drug-to-drug interaction. Thus, the present study mandates future investigations to define the roles of tissue selective PXR agonists in the RA inflammatory response.

Although we have not investigated directly whether solomonsterol A modulates NF-κB activities in the CAIA model, we have previously shown that this molecule has the potential to modulate this inflammatory mediator in a model of intestinal inflammation [21], suggesting that solomonsterol A might also ameliorate CAIA-induced arthritis through PXR-mediated NF-κB inhibition. However, considering that other pathways, distinct from PXR/NF-κB signaling, are also involved in the pathogenesis of CAIA and that solomonsterol A exerts a wide anti-inflammatory activity, additional molecular mechanisms need to be considered [19,21].

In conclusion, present results demonstrate that treatment with solomonsterol A, a marine natural PXR agonist, significantly attenuates arthritis development in CAIA mice harboring a humanized PXR. This PXR agonist reduces the degree of joint damage by inhibiting the expression of pro-inflammatory mediators. The data obtained in the present study support the conclusion that PXR activation exerts a beneficial effect in RA, given its anti-inflammatory properties, and could serve as a novel prophylactic modality for RA.

## Abbreviations

CDCA: chenodeoxycholic acid
*cyp3a11*: mouse cytochrome P450
FXR: Farnesoid-X-Receptor
GR: Glucocorticoid Receptor
IL-6: interleukin-6
IL-17: interleukin-17
IL-10: interleukin-10
INFγ: interferon gamma
LXRα: Liver-X-receptor alpha
MCP-1: monocyte chemoattractant protein-1
*mdr1*: multi-drug resistance 1
*mrp2*: multidrug resistance-associated protein 2
PPARγ: Peroxisome Proliferator-Activated Receptor gamma
PXR-LBD: Pregnane-X-Receptor-Ligand Binding Domain
TGF-β1: transforming growth factor beta
TNFα: tumor necrosis factor alpha
